# An *in vivo* reporter of BMP signaling in organogenesis reveals targets in the developing kidney

**DOI:** 10.1186/1471-213X-8-86

**Published:** 2008-09-18

**Authors:** Ulrika Blank, Marianne L Seto, Derek C Adams, Don M Wojchowski, Michele J Karolak, Leif Oxburgh

**Affiliations:** 1 Department of Molecular Medicine, Maine Medical Center Research Institute, 81 Research Drive, Scarborough, ME 04074, USA

## Abstract

**Background:**

Bone morphogenetic proteins (BMPs) regulate essential processes during organogenesis, and a functional understanding of these secreted proteins depends on identification of their target cells. In this study, we generate a transgenic reporter for organogenesis studies that we use to define BMP pathway activation in the developing kidney.

**Results:**

Mouse strains reporting on BMP pathway activation were generated by transgenically expressing β-galactosidase under the control of BMP responsive elements from *Id1*. Reporter expression corresponds well with immunoassays for pathway activation in all organs studied, validating the model. Using these reporters we have generated a detailed map of cellular targets of BMP signaling in the developing kidney. We find that SMAD dependent BMP signaling is active in collecting duct trunks, but not tips. Furthermore, glomerular endothelial cells, and proximal nephron tubules from the renal vesicle stage onward show pathway activation. Surprisingly, little activation is detected in the nephrogenic zone of the kidney, and in organ culture BMP treatment fails to activate SMAD dependent BMP signaling in nephron progenitor cells. In contrast, signaling is efficiently induced in collecting duct tips.

**Conclusion:**

Transgenic reporters driven by control elements from BMP responsive genes such as *Id1 *offer significant advantages in sensitivity and consistency over immunostaining for studies of BMP pathway activation. They also provide opportunities for analysis of BMP signaling in organ and primary cell cultures subjected to experimental manipulation. Using such a reporter, we made the surprising finding that SMAD dependent BMP signaling is inactive in nephron progenitors, and that these cells are refractory to activation by applied growth factors. Furthermore, we find that the BMP pathway is not normally active in collecting duct tips, but that it can be ectopically activated by BMP treatment, offering a possible explanation for the inhibitory effects of BMP treatment on collecting duct growth and branching.

## Background

Bone morphogenetic protein (BMP) signaling plays diverse and essential roles during development of numerous organ systems such as the heart [1-3], lung [4,5] and kidney [6-8]. Signaling is initiated when ligands bind cell surface serine threonine kinase receptors that in their turn phosphorylate the receptor associated transcription factors SMAD 1, 5 and 8 (R-SMADs) [9]. Phosphorylated R-SMADs associate with the common SMAD, SMAD4 and are translocated into the nucleus where they bind DNA in association with other factors. Transcriptional outcomes are determined by association with general transcriptional activators such as p300 and CBP [10], or by association with repressors such as Suv39h [11] or CtBP [12]. The SMAD Binding Element (SBE), GTCT and the GC-rich consensus motif GNCGCC [13-15], confer relatively low affinity and specificity to the SMAD:DNA interaction, necessitating association with other transcription factors such as ZFP423 (OAZ) [16] for efficient DNA binding. Interestingly, this combinatorial requirement for binding can be bypassed by concatamerizing the basic SMAD binding motifs. This approach has been successful in generating synthetic genes for use as signaling reporters in vitro [16,17]. The immediate early BMP response gene *Id1 *has been of particular interest as its promoter region contains a natural concatamer of SMAD binding motifs [15], potentially enabling its activation through SMAD binding alone. This natural concatamer, known as the BMP Responsive Element (BRE) has been used to generate both an in vitro BMP reporter [15] and in vivo reporters [18,19].

Although cellular targets for BMP signaling have been defined in some developing tissues by conditional inactivation of BMP receptors [20-25] and Smad transcription factors [26], the full complement of cellular targets of BMP signaling in many organ systems is not known. To further clarify which cells respond transcriptionally to SMAD mediated BMP signals during mouse organogenesis, we have generated a novel in vivo reporter by cloning a concatamer of the *Id1 *BRE upstream of the heat shock protein 1A (*Hspa1a *or *Hsp68*) promoter fused to β-galactosidase cDNA [27]. We find that β-galactosidase expression is regulated by BMPs in vitro, and can show by comparison with staining for nuclear phosphorylated SMAD 1, 5 and 8 that domains of BMP pathway activation can be recapitulated in all organs studied. In the kidney, we find that the BMP signaling pathway is highly activated in collecting ducts, developing nephron tubules and glomeruli, but surprisingly it is inactive in the nephron progenitor cell population, which is thought to respond to BMP signaling. Reporter gene activation is faithfully maintained in organ culture, confirming the versatility of this mouse strain for studies of organogenesis. Taken together, our study augments the current understanding of SMAD dependent BMP signaling during kidney development by identifying cellular targets.

## Results and discussion

### The BRE-Hspa1a-lacZ (BRE-lacZ) transgenic reporter strain

**Figure 1:**
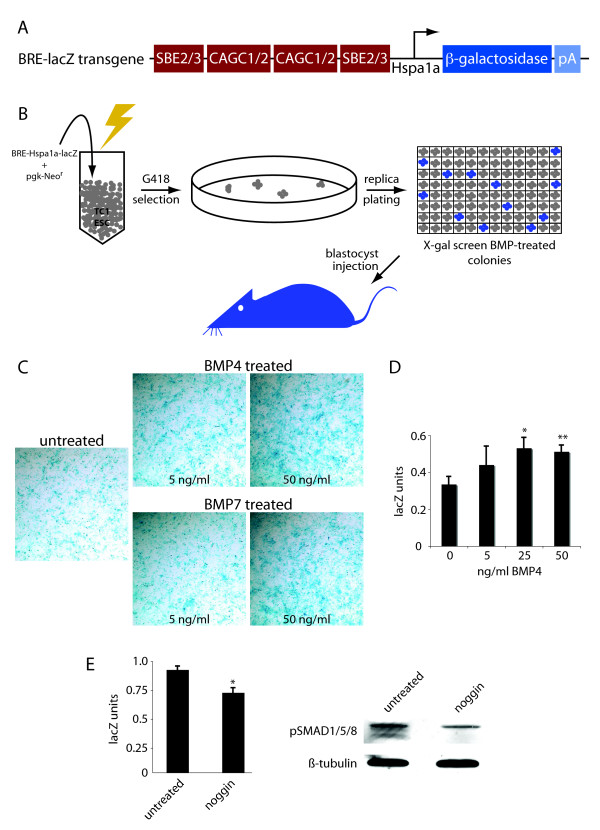
**Strategy for generating the BRE-lacZ reporter strain**. A. The BRE-lacZ transgene consists of a duplex of the CAGC1/2 sequence, and a duplex of SMAD binding elements 2 and 3 derived from the mouse *Id1 *gene [15] upstream of the *Hspa1a *promoter fused to β-galactosidase and the SV40 polyadenylation signal [27,28]. B. ES cell transgenesis was used to facilitate screening for signal strength of the reporter: ES cells were co-electroporated with BRE-Hspa1a-lacZ and a pgk-neo drug selection cassette, drug resistant colonies were isolated and screened for strong β-galactosidase expression in response to treatment with 25 ng/ml BMP4. Three clones were selected for blastocyst injection. C. Responsiveness of murine embryonic fibroblasts (MEFs) derived from BRE-lacZ positive E12.5 embryos was assessed by X-gal staining of cells. D. Responsiveness of MEFs to BMP treatment was verified by quantitative assay of β-galactosidase activity using the ONPG substrate. E. BRE-lacZ MEFs cultured in serum free medium for 48 hours with and without the addition of noggin were assayed for β-galactosidase activity using the ONPG substrate. For comparison of BMP pathway activation, cell extracts were immunoblotted for pSMAD1/5/8. Comparable loading of protein on the membrane was verified by immunoblot with β-tubulin.

To generate a mouse in which cells responding to BMP signaling are labeled by β-galactosidase expression, we derived a transgenic strain using a construct containing a concatamerized SMAD1/5/8 binding site from the mouse *Id1 *gene [15], upstream of the *Hspa1a *promoter (-873 to +5 relative to the translational start) fused to the β-galactosidase cDNA followed by an SV40 polyadenylation signal [27,28] (Fig. 1A). The BMP responsive cis-regulatory element has previously been successfully used to generate an in vitro BMP transcriptional reporter for use in cell culture [15], and an in vivo BMP reporter for early mouse development [18]. Both of these reporters employ the adenovirus major late promoter. For organogenesis studies, we chose the *Hspa1a *promoter as its high fidelity during development of diverse organ systems is well documented [29,30]. Furthermore, the Hspa1a-lacZ fusion construct has previously been successfully used to generate an in vivo reporter for retinoic acid signaling [31]. The *Hspa1a *promoter alone does not drive tissue specific expression, but does contain a heat shock element, causing ubiquitous activation of the reporter at 42°C [27]. Uniquely, therefore, the transgene can be heat induced in cells in which β-galactosidase is not expressed, to control for transcriptional silencing of the transgene.

To allow screening for transgenic reporters with high signal strength, we chose to derive transgenic mice from embryonic stem cells (ESCs) rather than using pronuclear injection. To evaluate the frequency in our experimental system of β-galactosidase activation caused by positional activation of the reporter gene, we performed a parallel control experiment in which Hspa1a-lacZ was electroporated into ES cells with the pgk-neo^r ^cassette, and drug resistant clones were assayed for reporter gene expression following BMP4 treatment. Of 96 drug resistant colonies screened, one was found to express β-galactosidase, indicating that the frequency of confounding constitutively β-galactosidase expressing clones was less than 2%. After selecting drug resistant ESCs coelectroporated with linearized BRE-Hspa1a-lacZ and selection cassette, individual colonies were screened for β-galactosidase expression in response to BMP4 treatment by X-gal staining (Fig. 1B). Approximately 13% (24/192) of drug resistant colonies expressed the reporter gene. As extensive BMP pathway activation is required for maintenance of undifferentiated ESCs [32], basal BMP reporter activation could not be entirely overcome. Therefore, it should be noted that colonies were selected on the basis of increase in X-gal staining relative to the unstimulated state. Three colonies with induced responses to ligand were selected for blastocyst injection, resulting in establishment of 2 lines derived from separate transgene integration events: 1C10 and 2F3. Sagittal sectioning with subsequent X-gal staining of E10.5 and E17.5 embryos revealed a highly similar β-galactosidase expression pattern in these two strains (See additional file 1: straincomparison.pdf). As the 1C10 strain displayed more intense staining, it was used for subsequent experiments, and is hereafter designated BRE-lacZ.

BMP inducibility of β-galactosidase expression in BRE-lacZ mice was verified by stimulation of murine embryonic fibroblasts (MEFs) with recombinant BMP4 and BMP7 (Fig. 1C, D). After stimulation with either of these ligands, β-galactosidase accumulates intracellularly in a dose-dependent manner. This can both be visualized by X-gal staining (Fig. 1C), and quantified by colorimetric assay using the ONPG substrate (Fig. 1D), demonstrating that the reporter is significantly activated by BMP4. As with ESCs, the basal level of reporter gene activation in MEFs is relatively high. This can be overcome to some degree by culture in serum free medium for 24 to 48 hours or addition of the BMP antagonist noggin to cell culture medium, but cannot be entirely quenched. A possible explanation for this residual basal pathway activation is that BMPs are actively produced in embryonic fibroblasts [33], and the extracellular antagonist noggin may be incapable of quenching signaling initiated intracellularly by BMP ligands. To explore this possibility, we compared lacZ production with the abundance of phosphorylated SMAD 1, 5 and 8 transcription factors (pSMAD1/5/8) in untreated and noggin treated BRE-lacZ fibroblasts (Fig. 1E). Although culture with noggin results in a significant reduction of both lacZ production and pSMAD1/5/8, basal activation does remain, demonstrating that extracellular BMP antagonism does not quench endogenous BMP signaling in fibroblasts. Previous experiments employing MEFs in which *Smad4 *had been conditionally inactivated demonstrated that TGFβ superfamily pathway activation is required for growth of these cells (data not shown and [34]), and thus basal pathway activation is anticipated in these actively growing cells.

From these experiments, we propose that domains of β-galactosidase expression correlate with BMP pathway activation in vivo rather than transgene activation due to positional integration effects as we find that transgenic animals generated from 2 separate ES cell clones display highly similar expression patterns. Furthermore, based on in vitro ligand stimulation assays we conclude that the BRE-lacZ transgenic strain expresses β-galactosidase in response to BMP.

### BRE-lacZ reporter activation correlates with nuclear accumulation of phosphorylated SMAD 1, 5 and 8

**Figure 2:**
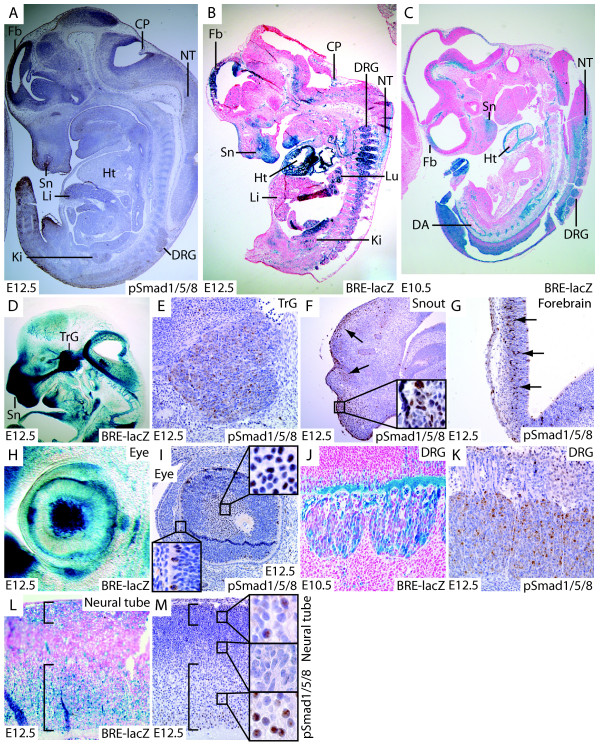
**Comparison of BRE-lacZ with nuclear accumulation of phosphorylated SMAD 1, 5 and 8**. A. In general, immunostaining for nuclear accumulation of phosphorylated forms of SMADS 1, 5 and 8 (pSMAD1/5/8) is weak, but strongest signals are seen in forebrain, snout, dorsal root ganglia, neural tube and kidney. B. At E12.5, intense reporter gene activation can be seen in areas overlapping with nuclear pSMAD1/5/8 staining: forebrain, snout, dorsal root ganglia, ventral neural tube, kidney and heart. C. At E10.5, reporter gene activation is intense in heart, forebrain, snout, neural tube, dorsal root ganglia and dorsal aorta as well as blood vessels of the head. D. Vibratome section of BRE-lacZ head showing strong signal in trigeminal ganglion (TrG) and snout (Sn). E. Abundant nuclear pSMAD1/5/8 is seen in the trigeminal ganglion. F. Nuclear pSMAD1/5/8 colocalizes with BRE-lacZ expression in the snout. G. Nuclear pSMAD1/5/8 in forebrain. H, I. Comparison between BRE-lacZ and pSMAD1/5/8 in the eye shows significant overlap in the corneal epithelial layer and lens (insets). J, K. β-galactosidase and nuclear pSMAD1/5/8 overlap in cells of the dorsal root ganglion. L, M. BMP signaling can be detected by both BRE-lacZ and pSMAD1/5/8 in the ventral and dorsal neural tube (square brackets, insets), but not in the intermediate region. Abbreviations: DA: dorsal aorta, CP: choroid plexus, DRG: dorsal root ganglion, Fb: forebrain, Ht: heart, Ki: kidney, Li: liver, Lu: lung, NT: neural tube, Sn: snout, TrG: trigeminal ganglion.

**Figure 3:**
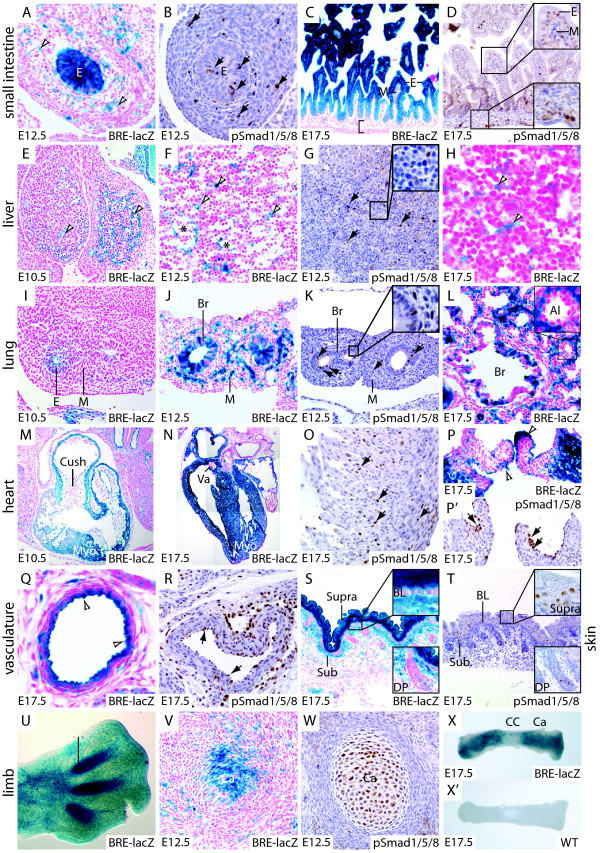
**Reporter gene activity in developing organs**. A – D. In the developing gut, reporter gene activity is seen throughout the gut epithelium at both E12.5 (A) and E17.5 (C). Nuclear pSMAD1/5/8 staining is seen in a subset of epithelial cells at E12.5 (B, arrows), and all epithelial cells at E17.5 (D). Scattered cells in the mesenchyme show reporter gene activation (A, open arrowheads), similar to the pattern of nuclear pSMAD1/5/8 staining (B, arrows). In the villus of the small intestine, the majority of mesenchyme cells show neither reporter gene activation (C) nor nuclear pSMAD1/5/8 (D, inset). Likewise, the gut wall is largely devoid of reporter gene activation and nuclear pSMAD1/5/8 (C, D, inset) with the exception of some blood vessels. E – H. In the developing liver, reporter activation is seen in endothelial cells of vessels (asterisk), and in individual cells distributed through the liver parenchyme (open arrowheads). G. A similar distribution of nuclear pSMAD1/5/8 staining is seen (arrows, inset). H. Expression is maintained in the liver through development to E17.5. I – L. In the lung, the reporter is active in large airway epithelium from E10.5 (I) to E17.5 (L). A mixture of activated and non-activated cells can be seen in bronchiolar epithelium (J, L), similar to the pattern of nuclear pSMAD1/5/8 staining (K, arrows, inset). In the E12.5 mesenchyme, sporadic reporter gene activation and nuclear pSMAD1/5/8 can be seen (J, K, arrows). In the E17.5 lung, no reporter gene activation is seen in alveolar epithelium, whereas widespread activation can be seen in the mesenchyme between alveoli (L, inset). M – P. In the heart, the reporter is active in myocardium from E10.5 (M) to E17.5 (N), overlapping with widespread nuclear pSMAD1/5/8 (O, arrows). In endocardial cushions (M) and developing semilunar valves (N), reporter activation is limited to endocardial cells (P, open arrowheads), overlapping with nuclear pSMAD1/5/8 (P', arrows). Q – R. In blood vessels, overlapping reporter activation and nuclear pSMAD1/5/8 are seen in endothelial cells (Q, arrowheads, R, arrows). S – T. In skin, the basal layer is devoid of both reporter gene activation and nuclear pSMAD1/5/8, whereas the suprabasal layer stains strongly for both (T, inset). The reporter is weakly active in the subbasal layer. Single cells within the base of each dermal papilla display both reporter activation and nuclear pSMAD1/5/8 (S, T, insets). U – X. In the developing forelimb, reporter activation is seen in the interdigital region of the E12.5 limb (U). Reporter activation and nuclear pSMAD1/5/8 overlap in cartilage of the developing humerus (V, W). In cultures of E17.5 metatarsals, reporter activation is seen in cartilage, but is excluded from the calcified center, despite treatment with 50 ng/ml BMP4 (X). X'. An X-gal stained wild type metatarsal demonstrates absence of background staining. Abbreviations: Al: alveolus, Br: bronchiole, BL: basal layer, Ca: cartilage, CC: calcified center, Cush: endocardial cushion, DP: dermal papilla, E: epithelium, IR: interdigital region, M: mesenchyme, Myo: myocardium, Sub: subbasal layer, Supra: suprabasal layer, Va: valve.

To definitively assay the degree to which in vivo reporter gene activation corresponds with pathway activation, we compared sections immunohistochemically stained for pSMAD1/5/8 with X-gal stained sections of BRE-lacZ embryos (Fig. 2). Nuclear pSMAD1/5/8 staining is a widely used marker of BMP pathway activation in diverse organ systems and species [35-37], reporting on activation of BMP-induced R-SMADs upon ligand stimulation. E12.5 was chosen as the most informative time point for these studies for two reasons. First, organogenesis is well underway, enabling comparison between nuclear pSMAD1/5/8 accumulation and reporter gene activation in most systems. Second, the paraformaldehyde fixed E12.5 embryo is readily sectioned, without tearing due to uneven fixation. Serial sagittal sections through 3 entire E12.5 wild type embryos were immunohistochemically stained with a polyclonal pSMAD1/5/8 antiserum (Cell Signaling Technology), and representative sections are shown (Fig. 2). Negative controls consisting of E12.5 wild type embryos stained either with secondary antibody alone, or with X-gal showed negligible background signal (See additional file 2: staining controls.pdf). Low power micrographs of medial E12.5 sections from wild type mice immunostained for pSMAD1/5/8 and X-gal stained BRE-lacZ reporters show extensive overlap in specificity of staining (Fig. 2A, B). Furthermore, the agreement in areas devoid of staining for both pSMAD1/5/8 and X-gal is notable in this overview, indicating that the reporter is sensitively and specifically reporting on BMP pathway activation, without spurious ectopic activation. Domains of reporter gene activation can also be seen at earlier developmental time points (Fig. 2C, additional file 1: straincomparison.pdf). Specifically, at E12.5 pathway activation with concomitant reporter gene activation is seen in the trigeminal ganglion (Fig. 2D, E), snout (Fig. 2D, F), forebrain (Fig. 2A, G), eye (Fig. 2H, I), dorsal root ganglia (Fig. 2J, K) and neural tube (Fig. 2L, M). One exception to the strong correlation between nuclear pSMAD1/5/8 staining and reporter gene activation is the embryonic heart, which does not show noticeable pSMAD1/5/8 staining in this assay. However, immunohistochemistry reveals relatively weak localized nuclear staining in this organ when fixed in isolation from the embryo (Fig. 3O). Furthermore, a previous study has reported nuclear pSMAD1/5/8 staining in cardiomyocytes of midgestation embryos [38], and we therefore conclude that the pathway indeed is activated in the heart, but that our immunoassay is not sufficiently sensitive for its detection.

Based on the comparison with nuclear accumulation of pSMAD1/5/8, we are confident that the BRE-lacZ faithfully reports on domains of BMP signaling in vivo.

### BRE-lacZ reporter gene activation in developing organ systems

To validate the reporter for organogenesis studies, we performed analyses on a variety of tissues at 3 distinct time points during development: E10.5, E12.5, and E17.5. Additionally, immunostaining for pSMAD1/5/8 was performed to allow correlation of pathway activation with reporter gene expression.

#### Gut (Fig. 3A–D)

At both E12.5 and E17.5, the reporter is active in the epithelium of the developing gut. In the mesenchyme, comparatively less staining is apparent, and at E17.5 few stained mesenchymal cells can be seen (Fig. 3C). Nuclear pSMAD1/5/8 is seen in a subset of epithelial cells at E12.5 (Fig. 3B), but in all epithelial cells at E17.5 (Fig. 3D). This staining pattern correlates well with previous reports, which have shown extensive nuclear accumulation of pSMAD1/5/8 in epithelial cells of the villus with absence of signaling in most of the villus mesenchyme [39,40].

#### Liver (Fig. 3E–H)

Two distinct populations of β-galactosidase positive cells can be seen from E10.5 through to E17.5: endothelial cells lining vessels, and single cells throughout the liver parenchyme. Nuclear accumulation of pSMAD1/5/8 is seen in a similar distribution (Fig. 3G).

#### Lung (Fig. 3I–L)

The reporter is active in epithelium and mesenchyme from E10.5 through E17.5. In bronchiolar epithelia, pathway activation is not uniform, with signaling and non-signaling cells interspersed (Fig. 3J, 3L). This pattern is reflected in our pSMAD1/5/8 staining (Fig. 3K), and in pSMAD1/5/8 staining previously reported [4]. In agreement with this, reporter gene activation strongly resembles the expression pattern of Follistatin like 1 (*Fstl1*, TGFβ stimulated clone 36), a BMP response gene [41]. Reporter gene activation in lung mesenchyme is not apparent at E10.5, but widespread at E12.5 and E17.5. Little reporter activity is seen in alveolar epithelia, whereas strong staining can be seen in the vessel rich mesenchyme between alveoli. Again, this pattern corresponds closely to previously reported pSMAD1/5/8 staining, and the expression pattern of *Fstl1*, which is localized mainly to endothelial cells and smooth muscle in the mesenchyme [41].

#### Heart (Fig. 3M–P')

A consistent pattern is seen from E10.5 (Fig. 3M) to E17.5 (Fig. 3N) (E12.5 not shown) with reporter activation in myocardium and endothelium, but with little or no activation in endocardial cushions and valves (Fig. 3P). E17.5 myocardium displays nuclear pSMAD1/5/8 staining (Fig. 3O), and a similar pattern has previously been reported in cardiomyocytes of the E11.5 heart [38]. Nuclear pSMAD1/5/8 accumulation in cardiomyocytes is not uniform in either of these studies, yet reporter gene activation does appear to be. Likely explanations for this discrepancy could be either that: i) The BMP pathway is indeed active in all cardiomyocytes, but our immunoassay is only sensitive enough to detect a subset of these, or ii) BMP signaling is cyclical in these cells, causing β-galactosidase to accumulate in the cell during a period of activation of the pathway, leading to staining in cells in which the pathway is inactive. Regardless of the explanation for this discrepancy, our data suggests that cardiomyocytes actively respond to BMP signaling on a population basis. BMP signaling is highly regulated in the endocardial cushions and valves of the developing heart, with few stained cells in the mesenchyme of either cushions (Fig. 3M) or semilunar valves (Fig. 3P). Reporter gene activation can however be seen in overlying endothelial cells (Fig. 3P'), which is consistent with the pattern of nuclear accumulation of pSMAD1/5/8. Consistent with our finding of strong BMP pathway activation in myocardium and weak or absent activation in endocardial cushions and developing valves, *Bmp2*, *4*, *5*, *7*, and *10 *are all expressed in a semi-overlapping pattern in myocardium [42-44], whereas the BMP signaling inhibitors *Smad6 *and *Smad7 *are vigorously expressed in endocardial cushions [20,45].

#### Vasculature (Fig. 3Q, R)

Reporter gene activation and nuclear accumulation of pSMAD1/5/8 are both primarily localized to endothelial cells, although there is some staining in vessel walls. Recent studies have shown that SMAD-dependent signaling in endothelial cells is stimulated by BMP9 and BMP10 ligands activating the orphan ALK1 receptor [46,47]. Furthermore, biologically active BMP9 is a circulating plasma component in the human [48], consistent with the luminal activation of BMP signaling seen in the reporter mouse strain.

#### Skin (Fig. 3S, T)

The reporter is differentially regulated in skin isolated from the lumbar region of the embryo: In the basal layer, little or no activation can be seen, whereas strong activation is seen in the suprabasal layer, and weak activation in the subbasal layer. Furthermore, individual cells within dermal papillae display activation. This data correlates well with a recent report, which suggested that BMP signaling is required in dermal papilla cells for their hair inductive properties [49]. Reporter activation is consistent with nuclear pSMAD1/5/8 accumulation, with the exception of the subbasal layer. As noted for other organs, the reporter is significantly more sensitive than the pSMAD1/5/8 immunoassay, and the lack of staining in the subbasal layer may be due either to weak SMAD expression, or to limited SMAD phosphorylation.

#### Limb (Fig. 3U–X)

The reporter is activated in the interdigital region of the E12.5 forelimb (Fig. 3U). Previous studies have identified a role for BMP signaling in apoptosis of the interdigital region [50], and this pattern of reporter gene activation is consistent with pSMAD1/5/8 nuclear accumulation in the developing autopod [51]. In the presumptive humerus at E12.5, strong reporter activation is seen in proximal and distal bone that contains resting and proliferative chondrocytes, while little or no activation is seen in the overlying perichondrium (Fig. 3V). Reporter gene activation correlates strongly with nuclear pSMAD1/5/8 in chondrocytes, and little or no staining in the perichondrium of the developing humerus (Fig. 3W). Strong nuclear accumulation of pSMAD1/5/8 has previously been observed in chondrocytes, and conditional inactivation of BMP type I receptors demonstrates that BMP signaling is required for cartilage condensation and skeletogenesis of endochondral bone [52]. Furthermore, compound inactivation of BMP2 and BMP4 indicate that these may be the cognate ligands required for BMP-mediated skeletogenesis [53]. To ascertain the degree of reporter activation in mineralized tissue, we cultured metatarsal rudiments from E17.5 BRE-lacZ embryos with or without 50 ng/ml BMP4 for 24 hours (Fig. 3X). The staining pattern is similar in both treated and untreated groups (data not shown), with strong signal in condensing or proliferative chondrocytes and a graded weaker signal toward the presumptive ossification center (Fig. 3X, X').

In this analysis of organogenesis of liver, gut, lung, heart, vasculature, skin, and bone, we find that there is a remarkable consensus between reporter gene activation and nuclear accumulation of pSMAD1/5/8. Furthermore, patterns of BMP reporter expression largely coincide with previous studies using the pSMAD1/5/8 antiserum. From this survey, we conclude that the BRE-lacZ strain is an appropriate tool for further detailed studies of organogenesis.

### Identification of BMP responding cells in the developing kidney

Numerous studies have defined roles for BMP ligands in development of the kidney. *Bmp7 *[7,8], *Bmp4 *[6] and *Bmp5 *[54] are all required for appropriate nephrogenesis. Interestingly, the molecular function of these ligands appears to be interchangeable [55], indicating that unique patterns of expression of these genes are decisive for development of the kidney rather than their individual properties [29,43]. Although detailed expression data is available for *Bmp *genes in the developing kidney [43,44,56], studies to define cellular targets of their gene products have generated ambiguous results, complicating the interpretation of loss of function phenotypes. This is presumably due to variability of reagents and protocols for detection [57-62]. Using the BRE-lacZ mouse, we are able to establish an unambiguous map of SMAD dependent BMP responses in the developing kidney, facilitating interpretation of loss of function mutants in the BMP pathway.

#### Mesonephros

**Figure 4:**
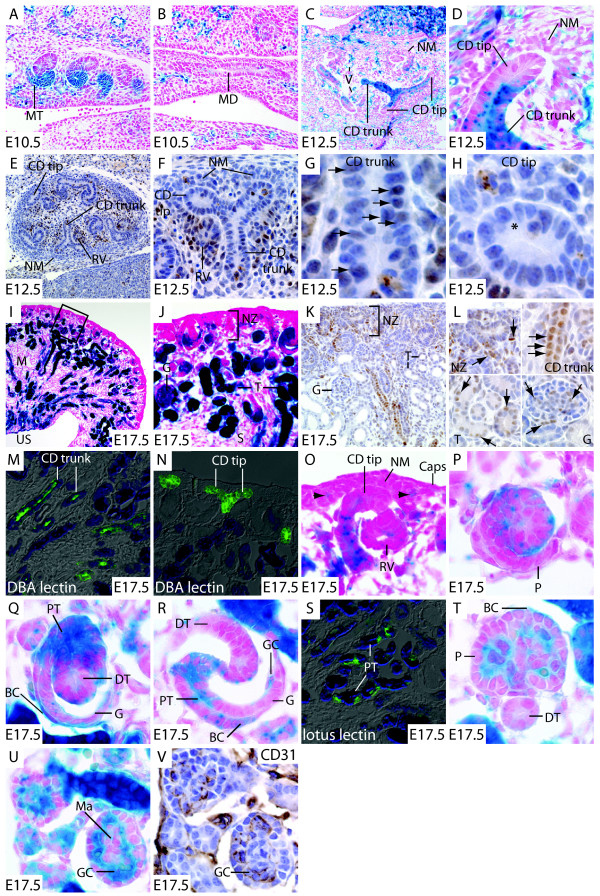
**BMP pathway activation in the developing kidney**. A. At E10.5, strong reporter activation is seen in mesonephric tubules within the intermediate mesoderm. B. Weak reporter activation is seen in the Wolffian duct at E10.5. C. At E12.5, the reporter is active in the trunk of the collecting duct, and islands of vascular activation can be seen throughout the mesenchyme. D. Collecting duct tips show no reporter activation. Likewise, nephrogenic mesenchyme shows little or no reporter activation. E. Nuclear pSMAD1/5/8 staining is seen in the center of the E12.5 kidney, with renal vesicles and collecting duct trunks showing the most intense staining. F. Very few cells interspersed within the nephrogenic mesenchyme display nuclear pSMAD1/5/8 staining. Cells of the renal vesicle, however display extensive nuclear pSMAD1/5/8 accumulation. G. Numerous cells within the collecting duct trunk display nuclear pSMAD1/5/8 staining (arrows). H. Little pSMAD1/5/8 staining is seen in collecting duct tips. I. Reporter activation is seen in tubules and glomeruli throughout the E17.5 kidney from the subcortical layer to the nascent papilla. J. Enlargement of the boxed region in panel D. Reporter activation is seen in tubules and glomeruli, but is largely absent from the nephrogenic zone. K. Nuclear pSMAD1/5/8 staining is seen in cells of the glomerulus, tubules and nascent nephrons. Nuclear pSmad1/5/8 staining is sparse in the nephrogenic zone. L. Enlargements of pSmad1/5/8 stained nephrogenic zone, collecting ducts trunks, nephron tubules, and glomerulus showing localization of nuclear pSmad1/5/8 accumulation (arrows). M. Costaining with DBA lectin shows reporter activation in collecting duct trunks. N. No activation can be seen in DBA staining collecting duct tips. O. In the nephrogenic zone, activation can be seen only in nascent nephron structures, and individual cells adjacent to the nephrogenic mesenchyme (arrowheads). P. Reporter activation can be seen throughout the early comma shaped body with the exception of the proximal podocytes. Q. At later stages in the comma shaped body, signaling is concentrated to the presumptive proximal tubule and Bowman's capsule. R. In the s shaped body, pathway activation is seen in the proximal tubule and Bowman's capsule. S. Costaining with lotus lectin shows that the pathway remains active in more mature convoluted proximal tubules of the medulla. T. Distal tubules characteristically located at the vascular pole of the glomerulus display little pathway activation. Cells of Bowman's display pathway activation, but podocytes do not. U, V. Adjacent sections from E17.5 BRE-lacZ embryos stained for reporter activation (U) and the endothelial marker CD31 (V) show pathway activation in the glomerular capillary. Mesangial cells display little activation. Abbreviations: BC: Bowman's capsule, Caps: Capsule, CD tip: Collecting duct tip, CD trunk: collecting duct trunk, DT: distal tubule, G: glomerulus, GC: glomerular capillary, MD: mesonephric (Wolffian) duct, M: medulla, Ma: mesangium, MT: mesonephric tubule, NM: nephrogenic mesenchyme, NZ: nephrogenic zone, P: podocyte, PT: proximal tubule, RV: renal vesicle, T: tubules, US: urinary space, V: vasculature.

In the E10.5 embryo, BMP signaling is activated in the proximal pole of the mesonephric tubule, and little or no activation can be detected in the distal pole, which connects to the mesonephric duct (Fig. 4A). This pattern of pathway activation corresponds closely with *Bmp7 *expression in these nephrons [56]. Uniform low level signaling is seen in the mesonephric duct (Fig. 4B). Little is known about the role of BMP signaling in development of the primitive kidney in the mouse. However, loss of function studies in the amphibian reveal a requirement for BMP signaling both for formation of the pronephric duct and tubule [63], indicating that activation of this signaling system may also be a requirement for pro- and mesonephros formation in the mammal.

#### Metanephros

Two developmental time points were chosen for studies of metanephros development: E12.5 and E17.5. At E12.5, the ureteric bud derived collecting duct migrates into the metanephric blastema and induces the nephron progenitor population, or nephrogenic mesenchyme. At this time signaling is most prominent in the trunk of the collecting duct, but not the tips or leading edges of these structures and little pathway activation can be seen in the nephrogenic mesenchyme (Fig. 4C, D). Within the blastema, islands of BMP-signaling vasculature can be seen. This pattern of signaling is corroborated by nuclear pSMAD1/5/8 accumulation (Fig. 4E, F, G, H) and previously published pSMAD1/5/8 immunostaining [61,62], showing pathway activation specifically in trunks but not tips of collecting ducts. In the E17.5 kidney, widespread activation of the pathway reporter is seen, with the exception of the nephrogenic zone (Fig. 4I, J). Tubular structures at numerous stages of differentiation and glomeruli display reporter activation. This pattern largely corresponds to nuclear accumulation of pSMAD1/5/8 (Fig. 4K, L), although pSMAD1/5/8 staining is weak in nephron tubules and in the glomerulus (Fig. 4L). To discern precise locations of pathway activation, we generated a series of high power micrographs, and performed molecular marker analysis for structures lacking distinct morphology.

#### Collecting ducts

To confirm that differential pathway activation in collecting ducts is maintained from E12.5 to E17.5, we counterstained X-gal stained kidney tissue from E17.5 BRE-lacZ embryos with the dolichos bifloris lectin, an established marker for collecting ducts. Signaling is maintained in collecting duct trunks from E12.5 to E17.5 (Fig. 4M, N), but collecting duct tips are devoid of signal at both of these time points. Interestingly, a requirement for BMP signaling for collecting duct morphogenesis has recently been revealed by tissue specific inactivation of the *Alk3 *BMP receptor gene with the collecting duct specific *Hoxb7cre *driver [25]. This contrasts with previous work in which it was shown that inactivation of the *Smad4 *signal transduction mediator with the same cre driver has little or no effect on kidney development [61]. This suggests that the BMP receptor may mediate collecting duct morphogenesis through SMAD independent signaling in collecting duct tips, possibly through activating the p38MAPK pathway. Signaling by BMP7 through this pathway has previously been demonstrated in cultured collecting duct cells. Interestingly, low doses of BMP7 stimulate morphogenesis of cultured collecting duct cells through the p38MAPK pathway, whereas high doses of BMP7 negatively regulate p38MAPK pathway activation in a SMAD dependent fashion [64]. The complex regulation of collecting duct morphogenesis by BMP signaling is underscored by the outcome of inactivation of *Alk3 *in this cell population: Compared to wild type mice, *Hoxb7cre*; *Alk3*^ca ^mice display an early increase in collecting duct branching followed by a reduction in growth and branching later in development [25].

#### Nephrogenic zone

The nephrogenic zone of the developing kidney is the region of de novo nephron formation, and is composed of peripheral stromal cells, nephrogenic mesenchyme (nephron progenitor cells), collecting duct tips and pretubular aggregates, the nascent epithelial aggregates that will give rise to nephrons (Fig. 4O). With the exception of a very small number of single cells in the vicinity of the nephrogenic mesenchyme, there is no pathway activation in this zone of the kidney (Fig. 4O). This is remarkable, considering that BMP signaling is required for survival of nephrogenic mesenchyme cells, and that *Bmp7 *null kidneys are prematurely depleted of nephron progenitors [8,55,65]. Furthermore, the *Bmp7 *loss of function phenotype has been correlated with the non-redundant expression of *Bmp7 *in the nephron progenitor population [43], indicating that active BMP signaling does indeed occur in the nephrogenic zone. Several alternative explanations to this apparent contradiction can be formulated. First, the *Id1 *reporter might be inactive specifically in the nephrogenic zone of the kidney, whereas other BMP responsive genes are not. To address this, we compared in situ hybridization assays for the known BMP responsive genes *Id1*, *Id3 *and *Bambi *in the Genepaint database (Genepaint.org, see additional file 3: ISH.doc). We find that none of these genes are expressed in the nephrogenic zone, whereas they are expressed in a pattern that appears to overlap with BRE-lacZ reporter activation in the medulla of the kidney. This strongly supports the contention that BMP signaling, rather than just *Id1 *expression is inactive in the nephrogenic zone. Second, BMP signaling may be mediated through SMAD independent rather than SMAD dependent signaling pathways, for example the p38MAPK pathway [64]. Third, BMP signals to the very small population of BMP responding cells adjacent to the nephrogenic mesenchyme may be required for maintenance of the nephrogenic mesenchyme. Fourth, BMP signals emanating from the nephrogenic mesenchyme may be required by a responding cell population outside the nephrogenic zone, such as developing nephrons, to maintain the nephrogenic mesenchyme. Fifth, BMP signaling may be required at a time point before establishment of the nephrogenic zone for survival of the nephrogenic mesenchyme population.

#### Developing nephrons

In the nascent renal vesicle, little pathway activation can be seen (Fig. 4O). Signaling becomes active throughout the early comma shaped body with the exception of the nascent podocytes (Fig. 4P), and this domain of pathway activation resolves to the presumptive proximal tubule section as the structure matures, with little signaling seen in the presumptive distal tubule. In the presumptive glomerulus of the late stage comma shaped body, signaling is seen in Bowman's capsule, but the podocytes remain devoid of activation (Fig. 4Q). This pattern of pathway activation is maintained in the s shaped body, where signaling can be seen in the proximal tubule, Bowman's capsule, and capillaries forming within the vascular cleft. The distal tubule and podocytes remain free from signaling (Fig. 4R). Costaining with lotus lectin demonstrates that BMP signaling is maintained in the proximal tubule as it matures (Fig. 4S). Little pathway activation can be seen in mature distal tubules (Fig. 4T).

*Bmps *are expressed in an overlapping manner at each stage of nephron development [43,56]: *Bmp7 *is expressed in the nephrogenic mesenchyme, and subsequently remains localized to distal tubules and podocytes. *Bmp2 *is first expressed in the renal vesicle, and subsequently becomes localized to distal tubules, whereas *Bmp4 *is expressed in the proximal tubule, and *Bmp3 *is expressed in Bowman's capsule. In synthesizing this data, we find no clear correlation between ligand expression and pathway activation in the nascent nephron. Other components of the pathway that have been studied provide limited insight into the pattern of activation. For example, SMAD4 is down regulated in condensing nephrogenic mesenchyme [59], providing a possible explanation for the lack of activation in this compartment. Also, the gene encoding the BMP antagonist USAG-1 is expressed in the developing distal tubule, potentially explaining the limited BMP activation in this segment of the nephron [66]. However, transcripts for the BMP type 1 and 2 receptors can be found throughout the developing nephron [67]. Counterintuitively, the BMP agonist crossveinless 2 is expressed in nephrogenic mesenchyme and regionally in the comma shaped body [68]. A caveat to these comparisons is of course that most expression data is based on transcript analysis, which does not necessarily correspond with production of active protein. Therefore, only negative in situ hybridization results constitute data that can be fruitfully interpreted. An in vivo reporter is thus an extremely valuable tool in designing further studies of the BMP pathway in nephron morphogenesis based on conditional gene inactivation.

#### Glomeruli

High power micrographs reveal that signaling is regionalized within the glomerulus. Podocytes display little or no signaling in contrast to cells of Bowman's capsule (Fig. 4T). To locate pathway activation in the core of the developing glomerulus, we performed X-gal and CD31 staining on adjacent sections from E17.5 BRE-lacZ kidneys (Fig. 4U, V). Staining for the CD31 endothelial cell marker overlaps with reporter activation, demonstrating that the pathway is activated in the glomerular capillary. Interestingly, presumptive mesangial cells display little pathway activation (Fig. 4U). Our findings correlate with the pattern of nuclear accumulation of pSMAD1/5/8 (Fig. 4L), and one previous study [69], in which it was furthermore shown that paracrine BMP signaling from podocytes to the endothelium significantly affects formation of the glomerular vasculature. *Bmp7 *is strongly expressed in the developing podocyte, but the early onset of aberrant development precludes detailed analysis of glomerulus formation in the *Bmp7 *loss of function mutant.

**Figure 5:**
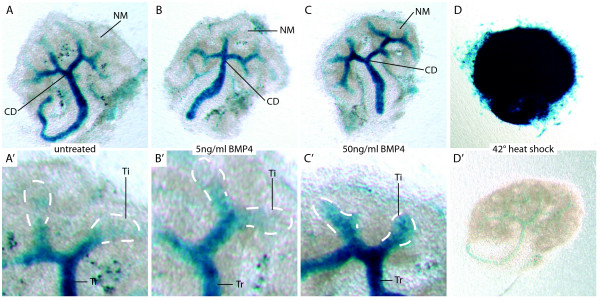
**Specific reporter gene activation is maintained in kidney organ culture supplemented with BMP4**. A – C. Strong reporter gene activation can be seen in collecting ducts of kidneys cultured for 48 hours in serum free medium with or without addition of BMP4, whereas the nephrogenic mesenchyme is devoid of reporter activation. A', B'. In untreated cultures, or cultures treated with a low dose of BMP4, reporter gene activation is limited to the collecting duct trunk. C'. At high dose of BMP4 treatment, the reporter is ectopically activated in collecting ducts tips. D. To control for the possibility that the transgene is inactivate specifically in the nephrogenic mesenchyme, transgene expression was activated by 120 minutes heat shock at 42°C. Strong expression of the transgene is seen throughout the organ. D'. Heat shocked wild type kidney explant demonstrating absence of background staining. Abbreviations: CD: collecting duct, NM: nephrogenic mesenchyme, Ti: collecting duct tip, Tr: collecting duct trunk.

As a first step toward understanding the molecular basis for requirement of BMP signaling in the developing kidney at the cellular level, we performed a series of organ culture experiments in which we treated explanted kidneys with recombinant BMPs. Considering the surprising lack of signaling in the nephrogenic zone, our primary question was whether this region is refractory to SMAD dependent BMP signaling, or whether it can be ectopically activated by growth factor treatment. In serum free culture conditions, addition of up to 50 ng/ml BMP4 to the culture medium does not cause reporter activation in the nephrogenic mesenchyme (Fig 5A–C), indicating that this cell population is indeed refractory to SMAD-dependent signaling. To control for the possibility that the reporter transgene might be transcriptionally silenced specifically in the nephrogenic mesenchyme, we performed an experiment in which transcription of the transgene was activated by heat shock to 42° for 2 hours. We find that heat shock activates transgene transcription in all cells of the explant, indicating that there is no confounding silencing of the transgene, and confirming that nephrogenic mesenchyme cells indeed are refractory to SMAD dependent BMP signaling (Fig. 5D, D'). Interestingly, collecting ducts do respond to increasing doses of BMP treatment by reporter activation in the tips (Fig. 5A'–C'). Studies have previously shown that BMP treatment of explanted kidneys causes inhibition of collecting duct growth and branching [65,70]. Cultures of collecting ducts in isolation have demonstrated that this is a direct effect rather than an effect mediated through the mesenchyme [71]. Our study indicates that high doses of BMP treatment function to ectopically activate SMAD dependent signaling specifically in the tip of the collecting duct, offering a possible explanation for the growth inhibitory function of BMP treatment. In support of this, low doses of BMP7 in culture have been proposed to stimulate growth and branching of collecting duct cells through activation of SMAD independent signaling pathways, whereas high doses inhibit growth and branching through activation of SMAD dependent signaling [64,70].

## Conclusion

The BRE-lacZ BMP reporter correlates well with immunoassays for pathway activation in the peripheral and central nervous system, gut, liver, lung, heart, vasculature, skin, developing bone and kidney, offering advantages in sensitivity and consistency over immunostaining for detection of pathway activation. A significant further advantage is the possibility to use the BRE-lacZ reporter in organ and primary cell culture experiments to explore pathway activation in response to experimental manipulation such as growth factor or chemical inhibition treatments. Using the reporter we have for the first time generated a detailed map of cellular targets of BMP signaling in the developing kidney, making the surprising finding that the nephrogenic progenitor cell population is refractory to SMAD dependent BMP signaling, and that the nascent nephron displays graded pathway activation. Furthermore, we find that the BMP pathway is not normally activated in collecting duct tips, but that it can be ectopically activated by treatment with recombinant BMPs, offering a possible explanation for the inhibitory effects of BMP treatment on collecting duct growth and branching. The molecular differences between nephrogenic mesenchyme and collecting duct cells, which most likely underlie their differential responses to BMPs constitute an interesting area for further research.

## Methods

### Transgenic reporter construct

To generate the BRE-Hspa1a-lacZ transgene, first, a pBS-BRE plasmid intermediate was created. The ~94 bp (Id1-BRE)_2 _enhancer fragment from the (BRE)_2_-luc plasmid [15] (kindly provided by Dr. Peter ten Dijke) was excised by NheI digestion and subcloned into SpeI linearized, dephosphorylated (Antarctic Phosphatase, New England Biolabs) pBS-KSII (Stratagene). Resultant clones were screened for insert by BssHII digestion. The pBShsp68lacZpA plasmid [28] (kindly provided by Dr. Brigid Hogan), was digested with SalI-XmnI and the 4.3 kb Hspa1alacZpA promoter β-galactosidase-polyA fusion was subcloned (1:1 insert:vector ligation) into SalI linearized and dephosphorylated pBS-BRE. A LacZ primer (5'-CCTGGAGCCCGTCAGTATCG-3') was paired with Stratagene T3 primer (5'-AATTAACCCTCACTAAAGGG-3') to screen resultant clones for orientation by whole cell lysate PCR. Clones with the BRE in the same orientation relative to the promoter as the original (BRE)_2_-luc were selected. For BRE-Hspa1a-lacZ, NotI – XmnI digestion was used to separate the 4.5 kb transgene from the vector backbone. To generate a neomycin resistance transgene, the 1.9 kb loxP-flanked pgk-neo^r ^gene was separated from vector backbone of the PL452 construct [72] (kindly provided by Dr. Neal Copeland) by EcoRI – BamHI digestion. Transgenes were gel purified, ethanol precipitated and resuspended in endotoxin free TE to 1 mg/ml for BRE-Hspa1a-LacZ and 0.5 mg/ml for pgk-neo^r^. Genotyping primers were designed to span the Hspa1a-LacZ junction: AC087117-F1 5'- TCTTGTCCATTCCACACAGG-3' and LacZ_1371R_J01636-1 5'- CTGCAAGGCGATTAAGTTGG-3', with an expected 622 bp product.

### Generation of transgenic mice

Animal care in accordance with the National Research Council Guide for the Care and Use of Laboratory Animals was approved by the Institutional Animal Care and Use Committee of Maine Medical Center. 20 μg of Hspa1a-lacZ or BRE-Hspa1a-LacZ and 5 μg of pgk-neo^r ^transgene were coelectroporated (500 μF, 0.24 kV, 9 msec pulse) into 8.24 × 10^6 ^TC1 ES cells (passage 11) in 0.5 ml PBS in a Bio-Rad GenePulser 0.4 cm cuvette. After a 5 minute incubation at room temperature, 1 ml of 37°C ES growth medium was added to each cuvette. ES growth medium consisted of HyQ DMEM/High Glucose (HyClone), 15% Embryomax ES cell fetal bovine serum (Millipore), 50 μg/ml gentamycin sulfate (Invitrogen), 0.1 mM beta-mercaptoethanol (Sigma) and 500 u/ml ESGRO (Chemicon). The resultant volume was divided into four 10 cm dishes of irradiated G418 resistant MEF feeder cells (passage 4) containing fresh 37°C ES growth medium. Twenty four hours later, medium was changed to G418 selective medium (300 μg/ml active concentration) in 3 plates, while 1 plate was changed to non-selective ES growth medium. Selective medium was changed every 1–2 days. After 10 days of G418 selection, 96 ES colonies with good morphology were picked from 2 selection plates for Hspa1a-LacZ, and 192 ES colonies were picked from three BRE-Hspa1a-LacZ selection plates. Colonies were picked in PBS containing calcium and magnesium, inoculated into plates containing 25 μl trypsin (Mediatech), incubated for 10 min at 37°C in a humidified CO_2 _incubator, interrupted by addition of 50 μl selective growth medium, triturated and replica plated in triplicate onto feeder cells (20 μl cells added to 175 μl selective growth media). After three days of growth, two of three replica plates were assayed for BMP responsiveness as follows. ES cells were serum starved for 2 hours, followed by BMP4 treatment (R&D systems, 0 or 20 ng/ml) in serum free medium for 16 hours. Plates were stained with X-gal according to routine procedures (0.5 mg/ml X-gal) and 24 clones from two 96-well BRE-Hspa1a-LacZ plates were selected based on positive staining for repeat analysis (0 or 50 ng/ml BMP4) with 16 hours of noggin pretreatment (100 ng/ml, R&D Systems). Three BRE-Hspa1a-LacZ lines were selected for robust response to BMP, expanded and used for C57BL/6J blastocyst injections. Chimeric founders were mated to ICR females and the F2 (ICRx129S6) progeny were used in this study.

### X-gal staining

E10.5 whole mount embryos and E12.5 vibratome sections were prepared and stained as described in [29]. For E17.5 and adult, individual tissues were cryosectioned and stained. Briefly, tissues were dissected and fixed in 1% formaldehyde, 0.2% glutaraldehyde for 30 minutes, then equilibrated in 30% sucrose overnight, embedded in OCT and sectioned at 5 microns. Sections were stained in 0.5 mg/mL X-gal overnight at 37°C.

### Immunohistochemistry

Immunohistochemical analysis was performed on 4% paraformaldehyde fixed paraffin-embedded tissue sections using antiserum specific for phosphorylated Smad1/5/8 (Cell Signaling Technology, 1:50 dilution). Following de-paraffination and hydration, sections were subjected to antigen unmasking by microwave heating for two cycles of 4 and 6 minutes in antigen retrieval solution (Dako Cytomation, 1:10 dilution). Sections were incubated at room temperature for 2 hours in the presence of primary antibody. Anti-rabbit HRP-conjugated secondary antibody (Amersham Biosciences, 1:500 dilution) was subsequently applied for 45 minutes at room temperature. Next, sections were incubated for 3 minutes with Biotinyl Tyramide Reagent according to manufacturer's protocol (Perkin Elmer). Vectastain (Vector Laboratories) was subsequently applied to sections for 30 minutes at room temperature. Stained cells were visualized using Sigma Fast 3,3'-diaminobenzidine (Sigma-Aldrich) according to manufacturer's instructions. Sections were counter-stained in Mayers Hematoxylin, and mounted (Cytoseal 60, Richard-Allan Scientific). CD31 staining was performed as previously described [59].

### Lectin staining

Frozen E17.5 kidney sections were X-gal stained as above for 30 minutes to 1 hour. Subsequently, sections were incubated at room temperature for 1 hour with either biotinylated lotus lectin (Vector Laboratories, Burlingame, CA, 1:200 dilution) or biotinylated dolichos biflorus agglutinin (Vector Laboratories, Burlingame, CA, 1:200 dilution). Following washing in PBS, sections were incubated with Streptavidin Alexa Fluor 488 conjugate (Molecular Probes, 1:600 dilution). Sections were mounted using Vectashield as above.

### Isolation and culture of murine embryonic fibroblasts

One litter of BRE-lacZ × ICR was harvested at E12.5. Following genotyping by X-gal staining of tails, 4 embryos of each wt and BRE-lacZ were dissected, removing internal organs and limbs. Fibroblasts were isolated by incubating minced embryos at 37°C for 10 minutes in 2 ml Trypsin-Versene solution (Cambrex Bioscience). Cells were brought into single cell suspension by trituration. Cells were plated and maintained in DMEM (Hyclone) supplemented with 10% bovine growth serum (Hyclone), 2 mM L-Glutamine (Invitrogen), 100 u/ml Penicillin (Sigma), 100 μg/ml Streptomycin (Sigma), and 2.5 μg/ml Amphotericin B (Sigma).

### BMP4 stimulation and quantification of β-galactosidase

Murine embryonic fibroblasts (MEFs) were seeded in 12-well plates at a density of 5 × 10^4 ^per well. 12 h post plating media was changed to DMEM supplemented with 10% serum replacement (Invitrogen), L-Glutamine, Penicillin, Streptomycin, and Amphotericin B. Following serum starvation for 36 hours, cells were stimulated for 16 h with 5, 25, or 50 ng/ml recombinant human BMP4 (R&D Systems). For analysis, cells were lysed using Reporter Lysis Buffer (Promega). Quantification of β-galactosidase in cell extracts was performed in 96-well format according to standard procedures [73] using 2-Nitrophenyl-β-D-galactopyranoside substrate (ONPG) (Sigma). Spectrophotometric readings were normalized for protein content of samples to control for differences in cell number between samples.

### Immunoblot

Total cellular proteins from MEFs, cultured under serum-free conditions as above with or without 200 ng/ml noggin (R&D Systems) for 48 hours, was extracted by boiling in sample buffer containing 4% sodium dodecyl sulfate (SDS), 120 mM Tris HCl pH 6.8, 180 mM dithiothreitol, 8.7% glycerol, and protease inhibitor according to the manufacturers' protocol (Roche). Proteins were separated by 8% SDS-polyacrylamide gel electrophoresis and transferred to a nitrocellulose membrane (Amersham Biosciences). Antibody specific for phosphorylated SMAD1/5/8 (Cell Signaling Technology) was used at 1/1000 dilution and anti-β-tubulin (Santa Cruz Biotechnology) was used at 1/3000 dilution.

### Organ culture

Kidney explants were cultured in serum free conditions as described in [74]. Following 24 hours culture, explants were treated either with no growth factor, 5 ng/ml, or 50 ng/ml recombinant human BMP4 protein (R&D Systems), and harvested for X-gal staining after 24 hours of stimulation. Metatarsals from E17.5 embryos were dissected free from surrounding tissues and cultured on Nuclepore filters (Millipore) floating on DMEM supplemented with Glutamine, Penicillin, Streptomycin, and Amphotericin B for 24 hours before X-gal staining.

## Abbreviations

BMP: bone morphogenetic protein; BRE: BMP responsive element; DMEM: Dulbecco's modification of Eagle's medium; ESC: Embryonic stem cell; lacZ: β-galactosidase; MEF: murine embryonic fibroblast.

## Authors' contributions

DCA performed the sectioning and X-gal staining, and performed and interpreted organ explant experiments. UB performed and interpreted the MEF assays, immunostaining and lectin staining experiments and helped draft the manuscript. MJK participated in the design and generation of transgene constructs. LO conceived of the study, performed and helped interpret the histological analysis and drafted the manuscript. MLS designed and built the BRE-lacZ transgene construct, generated the transgenic ES cells an helped draft the manuscript. DMW helped draft the manuscript.

## Supplementary Material

Additional file 1 Comparison of expression patterns of 2 BRE-lacZ reporter strains (1C10 and 2F3) derived from independent transgene integration events. Click here for file

Additional file 2 Negative controls for pSmad1/5/8 and X-gal tissue staining. Click here for file

Additional file 3 Links to Genepaint in situ hybridization results for BMP responsive genes *Id1*, *Id3 *and *Bambi*. Click here for file
