# Supplementary material for: An in vivo reporter of BMP signaling in organogenesis reveals targets in the developing kidney
Source: BMC Dev Biol. 2008 Sep 18;8:86. doi: 10.1186/1471-213X-8-86 (PMC2561030; doi:10.1186/1471-213X-8-86)
Supplement: Additional file 1 — Comparison of expression patterns of 2 BRE-lacZ reporter strains (1C10 and 2F3) derived from independent transgene integration events. [file 1471-213X-8-86-S1.pdf]

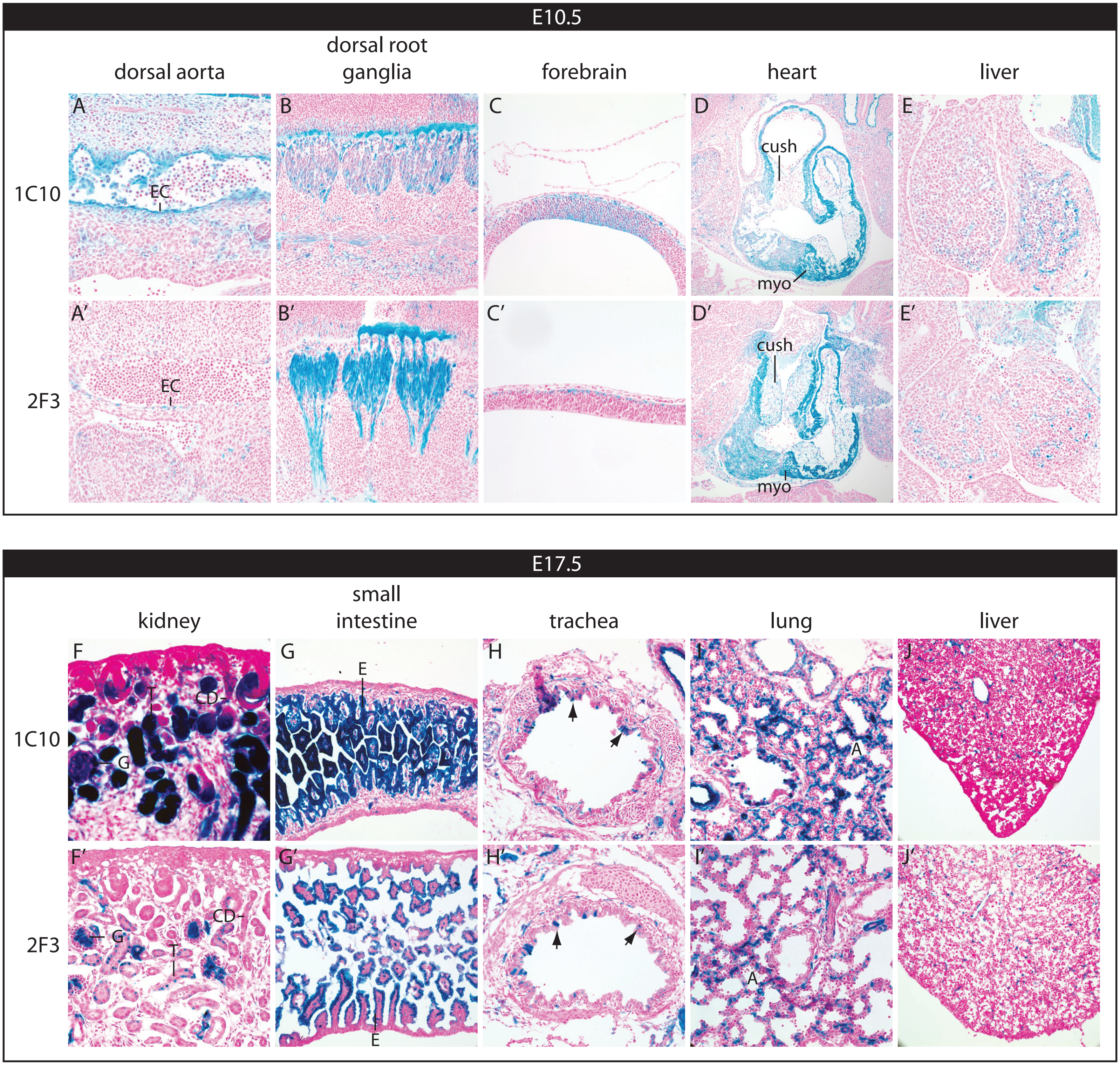

Supplementary Figure 1. β-galactosidase expression in two independent BRE-lacZ transgenic lines: 1C10 and 2F3. Comparison of 1C10 and 2F3 reveals highly similar expression throughout the embryo. Examples of staining at E10.5 in: A, A′. Endothelial cells (EC) of the dorsal aorta, B, B′. Trigeminal ganglia, C, C′. Forebrain, D, D′. Myocardium (myo), but not endocardial cushions (cush) of the heart, and E, E′. Liver. Examples of staining at E17.5 in: F, F′. Glomeruli (G), tubules (T), and collecting ducts (CD) of the kidney, G, G′. Epithelial cells (E) of the small intestine, H, H′. Cells interspersed within the epithelium of the trachea (arrows), I, I′. Cells in the mesenchyme between alveoli (A) of the lung, J, J′. Liver.
