# Supplementary material for: An in vivo reporter of BMP signaling in organogenesis reveals targets in the developing kidney
Source: BMC Dev Biol. 2008 Sep 18;8:86. doi: 10.1186/1471-213X-8-86 (PMC2561030; doi:10.1186/1471-213X-8-86)
Supplement: Additional file 2 — Negative controls for pSmad1/5/8 and X-gal tissue staining. [file 1471-213X-8-86-S2.pdf]

Negative controls for immunostaining and X-gal staining

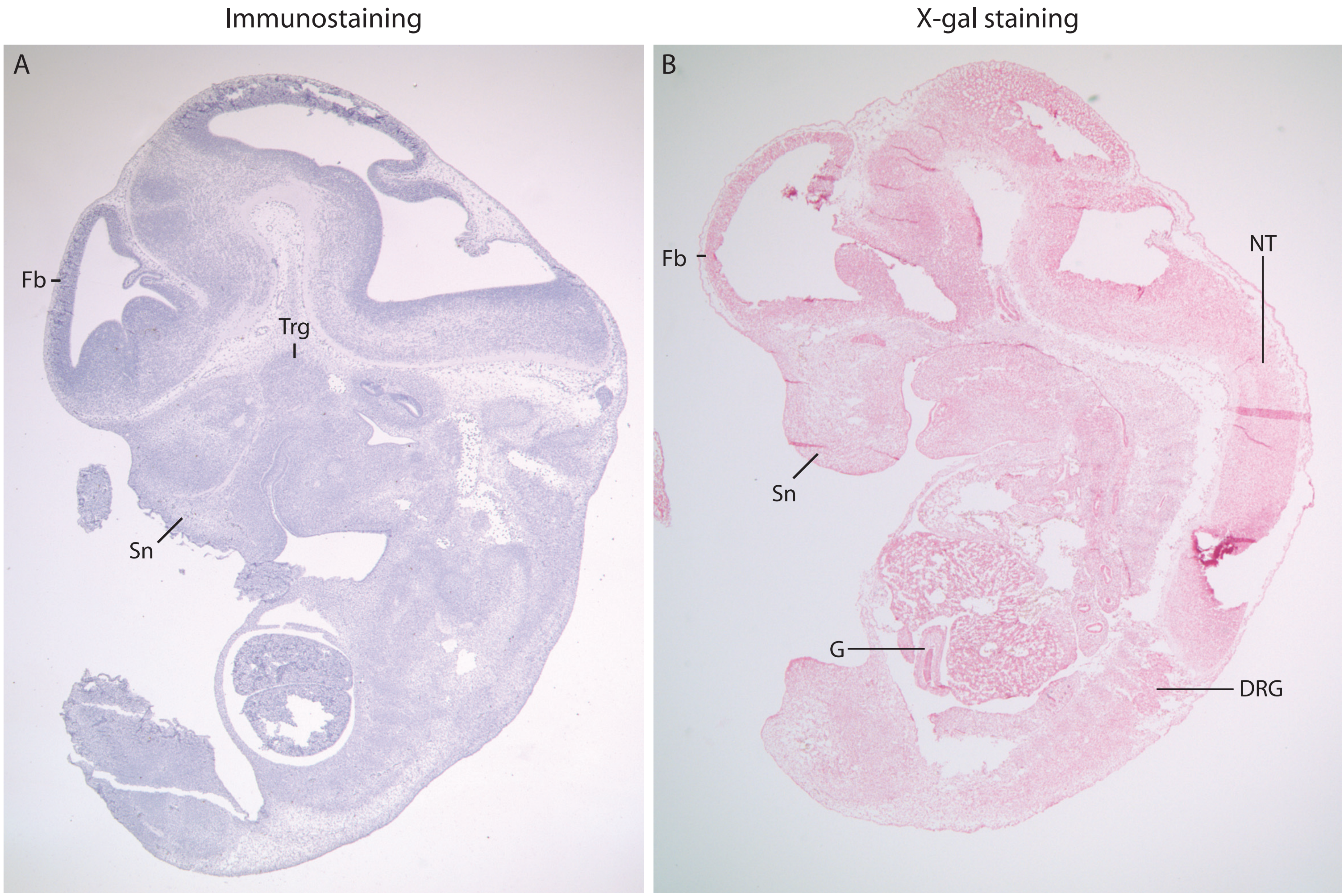

Supplementary Figure 2. Negative controls for pSmad1/5/8 immunostaining and X-gal staining.
A. Representative section from a series of 4 sagittal sections of E12.5 embryos processed simultaneously with pSmad1/5/8 stained sections shown in Figure 2. PBS was substituted for primary antibody. No staining can be seen in trigeminal ganglion (TrG), forebrain (Fb), snout (Sn), which display strong nuclear staining with pSmad1/5/8 antiserum. **B**. Representative section from a series of 12 sagittal sections of wild type E12.5 embryos stained with X-gal. No staining can be seen in any part of the embryo including forebrain (Fb), snout (Sn), gut (G), neural tube (NT) and dorsal root ganglia (DRG), which display strong staining in embryos of the BRE-lacZ genotype.
