# Supplementary material for: An in vivo reporter of BMP signaling in organogenesis reveals targets in the developing kidney
Source: BMC Dev Biol. 2008 Sep 18;8:86. doi: 10.1186/1471-213X-8-86 (PMC2561030; doi:10.1186/1471-213X-8-86)
Supplement: Additional file 3 — Links to Genepaint in situ hybridization results for BMP responsive genes Id1, Id3 and Bambi. [file 1471-213X-8-86-S3.pdf]

**Web addresses for Genepaint.org in situ results**

Id1
http://www.genepaint.org/cgi-bin/mgrqcgi94?APPNAME=genepaint&PRGNAME=analysis_viewer&ARGUMENTS=-AQ37501800418604,-AEB,-A215,-Asetview

Id3
http://www.genepaint.org/cgi-bin/mgrqcgi94?APPNAME=genepaint&PRGNAME=analysis_viewer&ARGUMENTS=-AQ37501800418604,-AEN,-A1225,-Asetview

Bambi
http://www.genepaint.org/cgi-bin/mgrqcgi94?APPNAME=genepaint&PRGNAME=analysis_viewer&ARGUMENTS=-AQ37501800418604,-AMH,-A230,-Asetview
